# Impact of Relationship Status and Quality (Family Type) on the Mental Health of Mothers and Their Children: A 10-Year Longitudinal Study

**DOI:** 10.3389/fpsyt.2017.00266

**Published:** 2017-11-29

**Authors:** Jasmin Hannighofer, Heather Foran, Kurt Hahlweg, Tanja Zimmermann

**Affiliations:** ^1^Department of Psychosomatics and Psychotherapy, Hannover Medical School, Hanover, Germany; ^2^Department of Clinical Psychology, University of Klagenfurt, Klagenfurt, Austria; ^3^Department of Clinical Psychology and Psychotherapy, University of Braunschweig, Braunschweig, Germany

**Keywords:** child emotional and behavioral problems, family type, unstable family structure, relationship quality, single mothers

## Abstract

Mothers and children of single or unstable relationships have higher rates of mental health problems than those in stable two-parent families. Despite results that mothers and children of conflictual two-parent families also show impairments, most studies do not consider relationship quality. Therefore, the present study combines relationship status and relationship quality to a “family type.” The present study compares German mothers and children of two-parent families with high relationship quality to those from two-parent families with a low quality, single mothers, and unstable families. Data of *n* = 249 families from a 10-year follow-up longitudinal study show that mothers with a high relationship quality show the highest levels of mental health whereas all other groups show at least a 3.2 times higher probability of mental health symptoms. Children of mothers in unstable relationships show a 8.2 times higher probability to emotional or behavioral problems than children of mothers with high relationship quality. Therefore, not only relationship status but also relationship quality should be combined and this “family type” should be considered in future research.

## Introduction

In the last half-century, structure and stability of families has changed dramatically. Due to increasing divorce rates and decreasing numbers of marriages worldwide, a growing number of children are raised in alternative family types (e.g., single-parent families or unstable parent relationships). In Germany, the number of single parents (SPs) increased from 14% in 1996 (1,304,000 single-parent families) to 20% in 2012 (1,607,000 single-parent families) (1). Previous studies showed that single mothers have considerably higher depression and anxiety symptoms compared to mothers of two-parent families (2–4). Moreover, children raised by SPs have a higher probability of developing mental health problems compared to children raised in two-parent families (2, 5–9). Results of the Millennium Cohort Study indicate that mothers and children who live in unstable families (separation, divorce) have more mental health problems than those of stable families (4, 10). Most of the previous studies focus on relationship status. They compare the differences between two-parent families and SPs or parents with relationships without taking the quality of relationship into account. Thus, it remains unclear if the presence or absence of a relationship is responsible for the rates of mental health problems or if the quality of the relationship is an important parameter. Currently, only few studies include relationship quality and compared mental health of mothers and children in stable but unhappy relationships with those of happy relationships. Few outcomes indicate that mothers and children of conflictual two-parent families have more mental health problems (9). Therefore, the current study tries to fill this gap. In the present study, which was conducted in Germany, 10-year longitudinal data were used to combine marital status (stable versus unstable) and relationship quality (high versus low). The new variable “family type” was created, resulting in four different types (stable families with high relationship quality, stable families with low relationship quality, unstable, and single families).

Children’s living arrangements have become increasingly diverse and instable, which raises important questions about how family structure and stability are related to mother and child outcomes. Several studies that focus on marital status found that married mothers had less mental health problems than unmarried mothers (11, 12) or single mothers (4). Butterworth (13) found approximately 45% of single mothers experienced depression or anxiety in the previous 12 months after separation compared to 23.6% of partnered mothers. Persons living in unstable relationships with no or changing relationships showed higher values in depressive symptoms and anxiety symptoms compared to persons in stable relationships (14). In addition, a correlation between a higher number of changes in relationship status and higher stress values were found (5, 15, 16). Crosier et al. (3) also examined the influence of different socio-demographic variables (mother’s education, employment, income, and age) on group differences. They found that single mothers were more likely than mothers in a relationship to live in a low socio-economic area, be unemployed, have lower household income, experience greater financial hardship, and have not completed 12 years of school. Single mothers were also more likely to be younger in age and have only one child. Different studies support these findings (14, 17).

Similar results were found in studies that examined the influence of relationship status of the mother on children’s mental health. Children of mothers in unstable relationships (5, 16) and children of single mothers (9) show a higher number of mental health problems compared to children of stable two-parent families. Schick (6) reported significant differences for mental health problems between children of divorced families and non-divorced families on the Child Behavior Checklist (CBCL) (18). Franz et al. (2) found that sons of single mothers had higher total scores and externalizing problems on the CBCL compared to partnered mothers.

Moreover, several studies found a positive correlation between the number of changes in relationship status (19–22) as well as type of changes (separation, divorce, or new partner) and mental abnormities in children (4). Children who experienced three changes in family status have a significant higher probability of developing externalizing and internalizing problems compared to children who do not experience a change (19). Cooper et al. (20) reported that not only the number of partners living together with the mother and the child but also the number of mother’s dating partners has a significant impact on externalizing problems in children. Most of the previous studies focus on relationship of mother and child and the influence of changing in family structure on mental health of both. To our knowledge, less is known about the father–child relationship.

Besides research results according to family status, some studies already have demonstrated the impact of relationship quality on mental health problems of mother and child. Low relationship quality is associated with a higher number of mental health problems (9, 23–26). Beach et al. (27) examined women who were unsatisfied with their marriage and depressive. In their marital discord model of depression, they describe that a decline in relationship quality comes along with an increase of depressive symptoms. A relationship with high quality, therefore, is a protective factor for the mental well-being of a married couple. Besides depressive symptoms, conflicts in relationship also correlate with the risk of developing substance-induced problems or anxiety symptoms.

In one of the few longitudinal studies that considered relationship and quality, Clavarino et al. (9) used data of an Australian longitudinal study and divided their sample into five groups at the 14-year follow-up: primary families with high relationship quality, primary families with low relationship quality, reconstructed families with high quality, reconstructed families with poor adjustment and unpartnered mothers (who were divorced, separated, widowed, or never married). Results at the 21-year follow-up indicate that mothers with low quality and also their young adults show significantly more often depressive symptoms than mothers and their children in high-quality relationships. A change from high to low quality and also to a single status correlates with an increase in depression.

While a change in family status often leads to a decrease of children’s mental health, mothers’ risk of depression decreases when a low-quality relationship ends. Children living in families with a high conflict rate have a lower mental well-being than children reared by parents with fewer conflicts (28–30). Based on the “Emotional Security Theory (EST),” Cummings and Davies (31) developed a process-oriented model to explain the correlation between parental conflicts and resulting behavioral problems of the child. They assume that chronic conflicts between parents not only insecure the parent–child relationship but also diminish children’s trust in parental ability to solve conflictual situations. Both increase the risk for mental health problems in children.

In sum, previous findings showed that mothers and children living in single or unstable relationships more often have emotional problems than those living in two-parent families. One reason could be that single mothers were more likely than mothers in a relationship to live in a low socio-economic area, to be unemployed, to have lower household income, to experience greater financial hardship, and to have not completed 12 years of school (3, 14, 17). The few studies that compared mothers and children living in stable but unhappy relationships with those living in relationships with high quality show that mothers and children of conflictual two-parent families have more mental health problems. But it is unclear, so far, to what extend a high relationship quality prevents mental health problems in mother and child and whether mothers and children living in a stable but low-quality relationships have the same risk of developing mental health problems as mothers and children of single or unstable relationships.

To answer these questions, we combined relationship status (yes or no) and relationship quality (high versus low) to a new term called “family type” which allows a conjoint consideration of two crucial factors on mother’s and child’s emotional and behavioral problems instead of analyzing these aspects separately. Mothers were divided into four different family types: SPs, mothers in with an unstable relationship history (UP), as well as mothers in two-parent families with low relationship quality (LQ) and high relationship quality (HQ). In detail, this long-term study aims to explore if these groups differ in terms of development of mental health problems in mothers and children over 10 years (1). We hypothesized that (2) mothers of two-parent families with high relationship quality (HQ) and their children (3) show a lower probability of developing mental health problems compared to single mothers (SP) and their children, mothers with low relationship quality (LQ) and their children as well as mothers and children of unstable relationships (UP). In addition, we hypothesized that (4) effects of hypotheses 2 and 3 were even significant higher if we controlled for group membership over 10 years of assessment, meaning that mothers and their children that had a high relationship quality at each time of assessment had a significant lower probability to become mentally ill compared to families with low relationship quality at each time of measurement.

## Materials and Methods

### Participants and Procedure

#### Sample

The sample was drawn from a German 10-year-follow-up study of the “Future Family Project” (32) which started in 2002 and was a randomized controlled universal prevention study for child behavior problems. Families were recruited from 17 preschools/kindergartens in Germany to participate in a randomized controlled trial of a parent training program (Triple P; 33). For the current analysis of the family type with four groups, a comparison between treatment and control group was not possible due to small sample sizes. We included Triple-P participation as a control variable. Participants in the Triple P group were offered four Triple P group sessions. They had to attend at least two sessions.

Parents fluent in German were eligible to participate if they had a child 3 to 6 years old attending preschool. The population response was 31% (*N* = 280) of those invited to participate, which is similar to other international prevention trials (33). Participants were assessed seven times over the course of the 10-year follow-up (pre; post-intervention, approximately 6 month following the initial assessment; four additional times every 12 month after the pre-assessment (follow-up 1–4); and follow-up 5, 10 years after pre). The retention rate was very high over the 10 years (92% post, 99% follow-up 1, 96% follow-up 2, 91% follow-up 3 and 4, and 89% follow-up 5). This study was approved by the human subjects’ research review board (German Society of Psychology; WS12_2010) and participants provided information consent.

At pre-assessment most of the families reported middle income (53%, 1,500 to 3,000 Euros per month); 34% reported higher income (>3,000 Euros per month), and 11% stated less income (<1,500 Euros per month). Employment characteristics were as follows: full-time salaried position or self-employment = 84.8% men, 15.7% women; part-time = 7.4% men, 46.3% women; stay-at home parent = 0.5% men, 30.6% women; and unemployed = 3.2% men, 2.9% women. Over half of the men and women (57 and 58%, respectively) had completed the high education level, 20% of men and 33% of women completed middle level, and 14% of men and 10% of women reported the low level of education. The average number of children living in the household was 2.1 (SD = 0.86) at pre.

At 10-year follow-up *n* = 249 families (89% of the original sample) could be reassess. The current sample consists of 131 (53%) boys and 118 girls. The mean age of children was 14.4 years (SD = 1.2, range 12–16 years; M_girls_ = 14.6, SD = 1.1, range 12–16; M_boys_ = 14.3, SD = 1.7, range 12–16). The majority of the participants were Caucasians (97%). The current age of the mothers was M = 48.8 years (SD = 4.7, range 33–57 years).

#### Change of Family Structure over Time

During the time since the child was born, family structure changed during the further course of study for most of the families. At first measurement point (when children were 3–6 years old), 88% of the parents were married or in a stable partnership, 8% of mothers were single, and 4% started a new partnership (unstable partnership). One year later at follow-up 1, only 79% of mothers still were in a stable partnership. Ten years after pre-assessment (follow-up 5), only 70% of the mothers still had the same partner.

At 10-year follow-up, only 97 of the 249 mothers still had the same relationship type as when the child was born. This includes those who were SPs for the whole time of child’s life (*n* = 18) as well as those who were with the same partner for the whole time since the child was born (*n* = 65), whereas some of these mothers were unsatisfied with their relationship for the whole time (*n* = 15) and most of them were happy with their relationship at each time of assessment (*n* = 50). Only few mothers (*n* = 14) got separated or divorced from the child’s father already during time of pregnancy and had at least one new partner at first measurement point (when child was between 3 and 6 years old). Mothers having an unstable relationship were younger [M = 43.8, SD = 4.6; *F*(3,244) = 11.72; *p* < 0.001] on average compared to mothers of the other groups. Children of single mothers on average were older [M = 14.9, SD = 1.3; *F*(3,245) = 11.72; *p* = 0.008] compared to other groups (see Table 1).

**Table 1:** Socio-demographic variables for SPs, unstable relationship (UP), low relationship quality (LQ) and high relationship quality (HQ) at 10-year follow-up.

	SP (*n* = 18)	UP (*n* = 76)	LQ (*n* = 60)	HQ (*n* = 94)	*F*	*p*
Mother’s mean age in years (SD, range)^a^^,^^b^^,^^c^	48.8 (6.7; 35–57)	43.8 (4.6; 34–53)	47.8 (4.6; 33–55)	45.6 (3.8; 34–55)	11.72	<0.001

Mother’s education level (*%*)^f^					0.70	0.55
Completing 9 or 10 years of school	8 (44.5)	38 (50.0)	23 (37.1)	36 (38.8)
Completing 12 or 13 years of school	9 (50.0)	34 (44.8)	39 (62.9)	57 (61.2)

Social status mother (*%*)^b^^,^^c^^,^^d^^,^^e^^,^^g^^,^^h^					9.40	<0.001
Low	3 (16.7)	9 (11.8)	1 (1.6)	-
Middle	8 (44.4)	29 (38.2)	13 (21.0)	27 (29.3)
High	6 (33.3)	37 (48.7)	47 (75.8)	62 (67.4)

Duration of mother’s relationship in years	–	–	24.1 (5.7; 13–35)	22.4 (4.7; 14–35)	3.59	0.06

Children’s mean age (SD, range)^b^	14.9 (1.3; 13–17)	14.7 (1.3; 11–17)	14.2 (1.1; 12–16)	14.3 (1.1; 12–17)	4.02	0.008

Child’s gender^b^					0.21	0.88
Female	10 (55.6)	35 (46.1)	28 (45.2)	44 (47.3)		
Male	8 (44.4)	41 (53.9)	34 (54.8)	49 (52.7)		

Number of siblings	1.4 (1.2)	1.5 (1.0)	1.1 (0.8)	1.5 (1.0)	1.00	0.36

*SP, single parent, UP, unstable relationship, LQ, stable relationship with low relationship quality, HQ, stable relationship with high relationship quality*.

*Low relationship quality = FBZ-K < 22; high relationship quality = FBZ-K ≥ 22*.

*^a^Significant differences between SP and IP (*p* < 0.001)*.

*^b^Significant differences between SP and HQ*.

*^c^Significant differences between UP and LQ*.

*^d^Significant differences between SP and LQ*.

*^e^Significant differences between UP and HQ*.

*^f^1 missing value for the SP sample; 4 missing values for the UP sample*.

*^g^Social status is indicated by the Winkler Index (34) (= monthly household income + school education + vocational education + recent profession)*.

*^h^1 missing value for the SP, UP, and LQ sample; 4 missing values for the HQ sample*.

#### Family Type

At 10-year follow-up categories were built for mothers and children of single households, mothers and children of unstable relationships, and of stable relationships (with high and low relationship quality) (see Table 1).

The group of *SP* (*n* = 18) included mothers who were single at all measurement points (pre-assessment to 10-year-follow-up) as well as mothers who were partnered but did not live together with their partner in one household. Hence, the main criterion for this group is whether mothers lived together with their partner or not. This classification corresponds to the definition of the Mikrozensus (35).

The group of *unstable relationship (UP)* (*n* = 76) included mothers with at least one change in relationship over the last 10 years. This group, for example, included mothers that were single at first measurement point and were partnered at one of the other measurement points or mothers that were partnered or even married at first measurement point and got separated and/or started at least one new relationship during time of the study.

The *stable group* with high or low relationship quality included mothers who were married or in cohabitation with the same partner since the child was born. To classify high versus low relationship quality the German version of the Abbreviated Dyadic Adjustment Scale (ADAS) (36, 37) was used. Quality of relationship was assessed six times over the course of the 10-year follow-up (five times in the first five years of assessment and one time 10 years after pre-assessment). To indicate the average quality of relationship over 10 years, a weighted mean index was calculated across all assessment points (for details, see Analytical Strategy). On the basis of the standardization of Sharpley and Rogers (36), mothers having an average value of 22 or higher over the course of 10 years were assigned to the high relationship quality group. Mothers having a value of 21 or lower were categorized to the low-quality group. Therefore, the following groups emerged: *SPs n* = 18, *unstable relationship (UP) n* = 76, *stable relationship with low relationship quality* (LQ) *n* = 62, and *stable group with high relationship quality* (HQ) *n* = 93.

### Measures

#### Child Behavior Checklist

The CBCL (18, 38) was used to assess emotional and behavioral problems of children. The CBCL 1½–5 (38) [German version (39)] consists of 100 items dealing with emotional and behavior problems of children age 1 1/2 to 5. The externalizing scale (Cronbach’s α in the current sample for mothers: α = 0.90) assesses conduct problems and the internalizing scale (α = 0.90 in the current sample) assesses withdrawal, depression, and anxiety. From the 2-year follow-up to the 10-year follow-up, the German version of the CBCL 4–18 (40) was used, which composed of 118 items to assess emotional and behavioral problems of children aged 4–18. The externalizing scale comprises items from the delinquent and aggressive behavior domains (α = 0.92 in the current sample), the internalizing scale comprises items from the withdrawn, somatic complaints and anxious/depressed scales (α: = 0.88/0.89 in the current sample).

#### Depression-Anxiety-Stress Scale (DASS)

The DASS (41) consists of 42 items, describing several psychological symptoms occurring in the last 4 weeks: 1 (*never*) to 4 (*very often*), whereas low values indicate low mental health problems. Answers can be summed up to three scales depression (α = 0.93 in the current sample), stress (α = 0.91 in the current sample), anxiety (α = 0.88 in the current sample), and a sum score (α = 0.96 in the current sample). In the current study, a German translation of the DASS (37) was used.

#### Abbreviated Dyadic Adjustment Scale

The German translation of the ADAS (36) called FBZ-K (37) was used to assess relationship adjustment (seven items: three items assessed topics of disagreement between partners, three items assessed frequency of positive exchanges, and one item assessed overall happiness). Items are scored on a Likert scale from 0 to 5, with higher scores indicating better relationship adjustment (α = 0.82 in the current sample). Values lower than 17 indicate a low relationship quality, values between 17 and 22 indicate an average quality and values from 22 and higher indicate a high relationship quality. In line with the standardization of the ADAS by Sharpley and Rogers (36), mothers with low and middle relationship quality were combined to the low relationship quality group for the current study. In addition, due to group sizes this decision was necessary.

### Analytical Strategy

A weighted mean index for the DASS and the CBCL was calculated across all assessment points indicating the average mental health of mother and child over 10 years of assessment: the first five measurement points together got the same weight as the last one because the first five measurement points were taken every year, the last one with a break of 5 years. In other words, the value of the last measurement point was added to the mean of the first five measurement points and accordingly divided by two. CBCL scores were *z*-standardized. Correlations were calculated between DASS and CBCL scores for the whole sample and for each group, separately. A binary logistic regression model was applied to study the impact of the diverse groups (SP, UP, LQ, and HQ) on the development of mental health problems in mothers (DASS) and children (CBCL). DASS and CBCL scores were included as dependent variables. Values in the upper quartile of the DASS and the CBCL scale indicate high mental health problems. The high-quality group (HQ) serves as reference group, because we expect that mothers and children of this group show lowest mental health problems. Therefore, we examined the influence of family type on mental health of mother and child for the whole sample. In addition, we controlled for demographic covariates, such as age and sex of the child, age of the mother, and the Winkler Index including income, school education, vocational education, and recent profession (34). Categories were “low,” “middle,” and “high.” In addition, we controlled for Triple-P participation (did parents take part into a parent training program or not).

As mentioned above, only 97 of the 249 mothers still had the same relationship type since their child was born. As a footnote analysis, we analyzed only these 97 families that could be assigned to one of the four groups since the child was born.

## Results

Table 2 shows means, SDs, and effect sizes for the DASS and the CBCL for the four groups of family type. Medium or large effects between the HQ group and all other groups (*d* = 0.47 to 0.56) were found for the DASS. We found no effects between the groups SP, UP, and LQ (*d* = 0.02 to 0.14). For the CBCL score, the effect sizes were highest for children in the HQ group compared to the other groups (internalizing: *d* = 0.22 to 0.31; externalizing: *d* = 0.28 to 0.30).

**Table 2:** Means, SDs, and effect sizes for scores on the Depression-Anxiety-Stress Scale (DASS) and Child Behavior Checklist (CBCL) for SP, unstable relationship (UP), relationship with low quality (LQ) and high quality (HQ) at mean of baseline to 10-year follow-up.

	SP (*n* = 18)	UP (*n* = 76)	LQ (*n* = 62)	HQ (*n* = 93)			
				
	M (SD)	M (SD)	M (SD)	M (SD)	Comparison	*d*^a^	*p*
DASS	22.4 (19.7)	24.5 (19.6)	22.0 (15.8)	15.5 (11.4)	SP-UP SP-LQ SP-HQ UP-LQ UP-HQ LQ-HQ	0.10 0.02 0.43 0.14 0.56*** 0.47***	0.99 0.97 0.11 0.99 <0.001 <0.001

CBCL internalizing	0.2 (1.2)	0.1 (1.2)	0.1 (0.8)	-0.2 (1.0)	SP-UP SP-LQ SP-HQ UP-LQ UP-HQ LQ-HQ	0.07 0.14 0.31^†^ 0.06 0.24^†^ 0.22	0.78 0.76 0.08 1.00 0.09 0.15

CBCL externalizing	0.2 (1.4)	0.1 (1.0)	0.1 (1.0)	-0.2 (0.9)	SP-UP SP-LQ SP-HQ UP-LQ UP-HQ LQ-HQ	0.06 0.06 0.29 0.01 0.28 0.30	0.81 0.93 0.19 0.97 0.25 0.13

*SP, single parent, UP, unstable relationship, LQ, stable relationship with low relationship quality, HQ, stable relationship with high relationship quality; M, Mean*.

*Low partnership quality = FBZ-K < 22; high relationship quality = FBZ-K ≥ 22*.

*All CBCL scales were z-standardized*.

^†^p < 0.10, ***p < 0.001.

*^a^*d* = effect size (Cohen): 0.20–0.39 small effect, 0.40–0.79 medium effect, and >0.80 large effect*.

**Figure 1:**
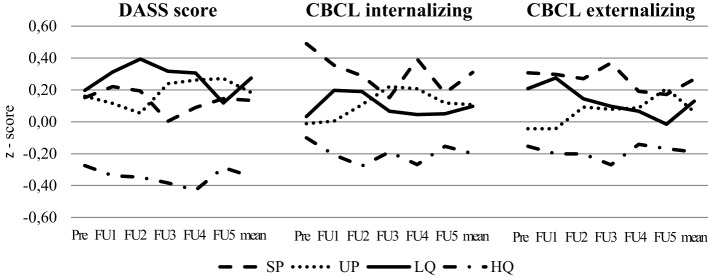
Changes of Depression-Anxiety-Stress Scale (DASS) scores and Child Behavior Checklist (CBCL) internalizing and externalizing score (*z*-standardized) from the first assessment point to 10-year follow-up.

Figure 1 demonstrates the average scores for the DASS and the CBCL over 10 years of assessment. Mothers in the HQ group have significant lower DASS values for assessment points three to five [*F*_FU3_(3,230) = 3.37; *p* = 0.019, *F*_FU4_(3,225) = 6.83; *p* < 0.001, *F*_FU5_(3, 223) = 3.6; *p* = 0.14], whereas single mothers (SP), mothers in low-quality relationships (LQ), and mothers with unstable relationships (UP) show the highest values. Regarding CBCL, a similar pattern can be found. Children of mothers of the HQ group show lowest values for internalizing problems [internalizing: *F*_FU3_(3,223) = 2.44; *p* = 0.065, *F*_FU4_(3,223) = 2.86; *p* = 0.035: externalizing: *p* > 0.05]. All other groups show similar courses over time.

As mentioned above, the analyzed sample was part of a parenting trial, but due to the small sample size, we decided to summarize control and treatment group and only to control for intervention. No significant differences between treatment and control group could be found for the DASS and CBCL.

### Differences between Mothers in High-Quality Group and Other Groups

First, we calculated correlations between mother’s mental health problems and children’s internalizing and externalizing problems for each group as well as for the whole sample. For each group and for the whole sample of *n* = 249, we found significant correlations between mother’s value of the DASS and internalizing as well externalizing problems of the child.

Using binary logistic regressions, we calculated two models (Tables 3 and 4). The first model included the family type and the DASS and CBCL index. For the second model socio-demographic variables (sex, children’s age, mother’s age, social status, and Triple-P participation) were added as control variables.

**Table 3:** Binary logistic regression analysis for the whole sample (*n* = 249) predicting influence of family type on mothers averaged weighted DASS (time: 1- to 10-year-follow-up).

	DASS^a^					
	Model 1			Model 2^b^		
	*n*	OR	95% CI	*n*	OR	95% CI
HQ	94	1 (Ref.)		91	1 (Ref.)	
SP	18	3.77*	[1.18, 12.09]	17	2.41	[0.61, 9.44]
UP	76	4.16***	[1.90, 9.12]	74	2.98**	[1.27, 6.95]
LQ	60	3.23**	[1.40, 7.47]	59	3.65**	[1.50, 8.87]
*R^2^* (Nagel-kerke)		0.09			0.19	

*SP, single parent, UP, unstable relationship, LQ, stable relationship with low relationship quality, HQ, stable relationship with high relationship quality; R^2^, explained variance by Nagelkerke R^2^; OR, odds ratio; CI, confidence interval*.

*^a^DASS: binary coded, values in the upper quartile (>0.42) or not. Model 1 χ^2^ (3) = 15.9, *p* < 0.001. Model 2 χ^2^ (9) = 33.3., *p* < 0.001*.

*^b^Model 2 (*n* = 241): added covariates: sex, children’s age, mothers age, social status, Triple-P participation*.

*p < 0.05, **p < 0.01, ***p < 0.001.

**Table 4:** Binary logistic regression analysis for the whole sample (*n* = 249) predicting influence of family type on children’s averaged weighted Child Behavior Checklist (CBCL) (Time: 1- to 10-year-follow-up).

	CBCL externalizing^a^					
	Model 1			Model 2^b^		
	*n*	OR	95% CI	*n*	OR	95% CI
HQ	94	1 (Ref.)		91	1 (Ref.)	
SP	18	2.27	[0.75, 6.89]	17	1.98	[0.45, 8.72]
UP	76	2.36**	[1.16, 4.78]	74	1.51	[0.69, 3.36]
LQ	60	1.25	[0.56, 2.81]	59	1.52	[0.64, 3.66]
*R^2^* (Nagel-kerke)		0.04			0.21	

**CBCL internalizing^c^**						

HQ	94	1 (Ref.)		91	1 (Ref.)	
SP	18	2.88*	[0.98, 8.52]	17	1.74	[0.48, 6.335]
UP	76	1.73	[0.84, 3.58]	74	1.29	[0.58, 2.87]
LQ	60	1.94*	[0.91, 4.16]	59	1.90	[0.85, 4.22]
*R^2^* (Nagel-kerke)		0.03			0.08	

*SP, single parent, UP, unstable relationship, LQ, stable relationship with low relationship quality, HQ, stable relationship with high relationship quality; R^2^, explained variance by Nagel-kerke R^2^; OR, odds ratio; CI, confidence interval*.

*^a^CBCL externalizing: binary coded, values in the upper quartile (>0.53) or not. Model 1 χ^2^ (3) = 6.7, *p* = 0.08. Model 2 χ^2^ (9) = 35.9, *p* < 0.001*.

*^b^Model 2 (*n* = 241): added covariates: sex, children’s age, mothers age, social status, Triple-P participation*.

*^c^CBCL internalizing: binary coded, values in the upper quartile (>0.31) or not. Model 1 χ^2^ (3) = 5.3, n.s. Model 2 χ^2^ (8) = 13.78, n.s*.

^†^p < 0.10, *p < 0.05, **p < 0.01.

As can be seen in Table 3, single mothers have a 3.8 times higher probability, mothers of unstable families a 4.2 times and mothers of the LQ group a 3.2 higher probability to reach values in the upper quartile of the DASS compared to mothers of the HQ group. Mothers of the SP, the UP, and the LQ group have higher values compared to the HQ group [*F*(3,243) = 4.50; *p* = 0.004], but do not differ from each other [*F*(2,173) = 0.87; *p* = 0.42]. By family type (model 1) *R*^2^ = 0.09 (Nagelkerke) of the variance of the mental health problems of the mother was explained. Controlling for socio-demographic variables such as age of mother and child, sex of child, social status, and Triple-P participation (model 2) mothers of unstable relationships (2.9 times) and mothers with low relationship quality (3.7 times) still have a significant higher probability to have mental health problems compared to mothers of the HQ group. Model 2 explains *R*^2^ = 0.19 (Nagelkerke) of the variance.

For the CBCL scores (Table 4), only children of the LQ group have a 1.9 times higher probability of developing internalizing and a 2.4 times higher probability of developing externalizing problems compared to children of the HQ group. Including socio-demographic variables, no significant differences were found.

### Regression Analysis of Families with the Same Family Type over 10 Years

Calculating binary logistic regressions for the 97 families who had the same family type over 10 years of assessment, single mothers have a 5.8 times higher probability, mothers of unstable families a 11.5 times and mothers of the LQ group a 10.1 higher probability to reach values in the upper quartile of the DASS compared to mothers of the HQ group. Controlling for socio-demographic variables (model 2) mothers of unstable relationships (7.0 times) and mothers with low relationship quality (10.2 times) still have a significant higher probability to have mental health problems compared to mothers of the HQ group. For the children, a significant higher probability of developing externalizing problems was found for the UP group (Model 1: 8.2 times higher, Model 2: 5.6 times higher).

## Discussion

The aim of this longitudinal study was to combine family status with relationship quality to establish a “family type” and to examine the impact of this family type on the mental health of mothers and their children over the long period of 10 years. The main finding of our study was that the family type (family status combined with relationship quality) emerged as a stronger parameter than only the relationship status (presence or absence of a relationship). The relationship quality seems to be the determining factor that has a long-term effect on mothers and children’s mental health, whereas some factors that are associated with improved mental health for mothers, do not have the same consequences for the children.

Most of the previous studies only compared mental health of mothers having different family status and found that married mothers and their children are happier and healthier than those of single or unstable families (4, 11, 14, 26). Living in an unstable relationship is a higher risk factor for mental health problems than living in a stable relationship anyway (not taken into account if mothers are happy or unhappy with their relationship) (19–21). Results of our study confirm results of the few studies that also included relationship quality and compared mental health of mothers and children in stable but unhappy relationships with those of happy relationships. Living in an unhappy relationship could have the same effect on mental health of the mother than living in an unstable relationship. In detail, our results showed that single mothers have a 3.8 times higher probability, mothers of unstable families a 4.2 times, and mothers of the low-quality group a 3.2 higher probability to reach values in the upper quartile of the DASS compared to mothers of the high-quality group. But the important result seems to be that these three groups do not differ from each other. That means, living in an unhappy relationship could lead to the same extend of mental health problems than living in an unstable relationship. Analysis of the 97 families with the same family type over 10 years also supports these findings. According to our results not the status of relationship but the quality of relationship seems to be the determining factor for the risk of mother’s mental health problems. With regard to the children, our results also confirm previous findings in some way. Only children of the low-quality group have a 1.9 times higher probability to develop internal and a 2.4 times higher probability to develop external problems compared to children of the high-quality group. Analyzing only the 97 families who had the same family type over 10 years of assessment, we found a higher probability to develop external problems (delinquent and aggressive behavior). And these results only were significant for the children in an unstable relationship. According to the results of the 97 families, it seems as if children are most infected by instability of relationship. One explanation could be that such a change in life is more incisive than fighting parents. According to the results of the whole sample, especially children of the low quality are affected by negative communication and conflict situations between parents. Being raised in a high-quality relationship or by a stable SP seems to be the most protective situation for the child, whereas for mothers only living in a positive relationship has a protective function. For mothers with a low-quality relationship, mental health problems are no worse if the mother decides to bring the relationship to an end.

### Strengths of the Study

A notable strength of the current study is the very high retention rate (89% at 10-year follow-up). Another advantage of the present analysis is the inclusion of comprehensive measures of sex of child, age of mother and child, income, and social status. Including additional predictors, such as the Winkler Index, sex, age of the child as well as mother’s age increases explained variance on average by 5%. Nevertheless, it should be assumed that other variables also have an influence on the variance, e.g., intensity of conflicts and also the social support by the father after breaking up (6). Moreover, Cina and Bodenmann (30) showed that parenting also explains part of the variance.

### Limitations of the Study

There are some limitations of the current study. First, on the basis of this study, the “chicken-and-egg-problem” could not be answered. We do not know if mental health problems of mother or child rather implicate a single or unstable family status or leads to a decline of relationship quality or if an inappropriate family status causes mental health problems. A combination of both ways seems to be plausible. Second, the German sample was part of a parenting trial, but due to small sample size we summarized treatment and control group. This is also an important consideration for generalizability of the findings. There were few differences between treatment and control participants in study variables, but notably women who received the parenting treatment did show fewer declines in relationship adjustment over time. We controlled for treatment conditions in all analyses, but it is possible that there may be further effects that we did not account for (e.g., interactions). Moreover we only controlled for the following demographic covariates: age of the child, sex of the child, age of the mother, the Winkler Index including income, school education, vocational education, and recent profession (34), and Triple-P participation (did parents take part into a parent training program or not). It could be mentioned that there are other variables to control for, but we tried to concentrate on the most important ones.

In addition, due to small sample size we assigned all mothers to the unstable group that changed their status within the course of 10 years of assessment, regardless of number of change, type of change or the duration of mothers’ relationship. The third limitation is given by the methodological procedure. During the follow-up assessments in this study different age-appropriate versions of the CBCL (CBCL 1½–5 and CBCL 4–18) were used. Although both versions are not completely equal, values were *z*-standardized. Fourth, the definition for single mothers is very conservative. Following the United State Census (42), mothers are categorized as single mothers when they do not live together with their partner. Osborne and McLanahan (22) and also Cooper et al. (20), however, showed that having a partner, also if he does not live together with the family, has an impact on mental well-being of the children. Contrary to this, Ständer et al. (7) found no differences in mental well-being for mothers and children regardless if a partner does exist or not. Unfortunately, the sample of single mothers was too small (*n* = 18) to investigate this potentially influence in this study. Fifth, according to Cooper et al. (20) who reported that not only the number of partners living together with the mother and the child but also the number of mother’s dating partners has a significant impact on externalizing problems in children, our sample size was too small to take this examination into account. Sixth, our study only describes the relationship between mother and child. For future studies, it would also be important to examine the father–child relationship. In the current study, we also did not consider to what extent the father takes care of the child after parent’s break up. Several studies showed that a high social support of the father comes along with low mental health problems (6, 43). Hence, this fact could have an immense impact on mental well-being. Seventh, the total amount of explained variance is not that high. One reason could be that a number of other factors that we have not measured play into the results.

### Conclusions and Implications

Especially due to the fact that an increasing number of children are reared in alternative family forms (1), this study compared continuously single-parent families, with families with unstable structures, and with intact families in which parents as couples have either high or low marital relationships. The results of the study show that despite the often repeated assertion that children of divorced and SPs are at risk for problems, the risk is similar to that for children of parents who stay together, but have a poor relationship. Considerable differences were found between mental health problems of mothers with high and low relationship quality and also for their children. Mother’s relationship satisfaction also seems to have a positive impact on children and keeps number of mental health problems low. Mothers living in unsatisfied relationships often ask themselves if they should leave the father or stay with him for the sake of the child. However, children may perceive the absence of a father more negative than living with unsatisfied parents. Living in a low-quality relationship has nearly the same effect on the mental well-being of children and mothers as living as a single or in an unstable relationship, but, overall, a child benefits from a healthy and satisfied mother. Couples with relationship distress may benefit from prevention and intervention programs. Prevention or relationship enhancement programs [e.g., Prevention and Relationship Enhancement Program in the US (44) or EPL in Germany (23, 45)] that lead to a positive and satisfied relationship could help parents to work on their relationship and to prevent mental health problems in individuals. Long-term impacts of mother’s mental health problems should not be underestimated (46). Furthermore, the results of the present study lead to the conclusion that changes in family status does not have more negative consequences than living in a stable but unsatisfied relationship. This should be confirmed in further investigations.

## Ethics Statement

The study was proved by the Ethics committee of the German Society of Psychology, WS12_2010.

## Author Contributions

JH, KH, and TZ have been involved in the development of the design. JH did the data collection. JH, HF, KH, and TZ have been involved in data analysis and the preparation of the manuscript. All authors contributed to the interpretation of the data. All the authors have read and approved the final manuscript.

## Conflict of Interest Statement

The authors declare that the research was conducted in the absence of any commercial or financial relationships that could be construed as a potential conflict of interest.

## References

[1] Statistisches Bundesamt. Familien mit minderjährigen Kindern nach Familienform. [Family Profiles for Families with Minor Children]. Wiesbaden: Statistisches Bundesamt (2013).

[2] Franz M Lensche H Schmitz N Psychological distress and socioeconomic status in single mothers and their children in a German city. Soc Psychiatry Psychiatr Epidemiol (2003) 38:59–68. 10.1007/s00127-003-0605-8 12563547

[3] Crosier T Butterworth P Rodgers B. Mental health problems among single and partnered mothers. The role of financial hardship and social support. Soc Psychiatry Psychiatr Epidemiol (2007) 42(1):6–13. 10.1007/s00127-006-0125-4 17203237

[4] Kiernan KE Mensah FK Partnership trajectories, parent and child well-being. In: Hansen K Joshi H Dex S, editors. Children of the 21^st^ Century: The First Five Years. Bristol: The Policy Press (2010). p. 77–94.

[5] Beck AN Cooper CE McLanahan S Brooks-Gunn J. Partnership transitions and maternal parenting. J Marriage Fam (2010) 72:219–33. 10.1111/j.1741-3737.2010.00695.x 21423848 PMC3057222

[6] Schick A Behavioral and emotional differences between children of divorce and children from intact families: clinical significance and mediating processes. Swiss J Psychol (2002) 61(1):5–14. 10.1024//1421-0185.61.1.5

[7] Ständer D Kuschel A Heinrichs N Bertram H Naumann S Hahlweg K Der Einfluss von Familientyp und Partnerschaftsqualität auf die psychische Situation von Müttern und Kindern. [Impact of family type and partnership quality on mental problems of mothers and children]. Psychol Erzieh Unterr (2007) 54:236–47.

[8] Robert Koch-Institut. Lebensphasenspezifische Gesundheit von Kindern und Jugendlichen in Deutschland. Ergebnisse des Nationalen Kinder- und Jugendgesundheitssurveys (KiGGS). Bericht für den Sachverständigenrat zur Begutachtung der Entwicklung im Gesundheitswesen. [Results of the National Child and Youth Health Survey (KiGGS): Life Stage Specific Health of Children and Adolescents in Germany]. Berlin: Robert Koch-Institut (2008).

[9] Clavarino A Bor W Hayatbakhsh MR O’Callaghan M Najman JM Williams GM. Depression following marital problems: different impacts on mothers and their children? A 21-year prospective study. Soc Psychiatry Psychiatr Epidemiol (2011) 46:833–41. 10.1007/s00127-010-0253-8 20574844

[10] Baldridge S Family stability and childhood behavioral outcomes: a critical review of the literature. J Fam Strengths (2011) 11(1):1–24.

[11] Meadows SO McLanahan SS Brooks-Gunn J. Stability and change in family structure and maternal health trajectories. Am Sociol Rev (2008) 73(2):314–34. 10.1177/000312240807300207 20333277 PMC2843941

[12] Joshi H Hansen K Dex S Introduction. In: Hansen K Joshi H Dex S, editors. Children of the 21^st^ Century: The First Five Years. Bristol: The Policy Press (2011). p. 1–11.

[13] Butterworth P. Lone mothers’ experience of physical and sexual violence: association with psychiatric disorders. Br J Psychiatry (2004) 184:21–7. 10.1192/bjp.184.1.21 14702223

[14] Boyle M Cairney J Offord DR Racine Y. Stress, social support and depression in single and married mothers. Soc Psychiatry Psychiatr Epidemiol (2003) 38:442–9. 10.1007/s00127-003-0661-0 12910340

[15] Reichman NE Teitler J Garfinkel I McLanahan SS Fragile families: sample and design. Child Youth Serv Rev (2001) 23(4/5):303–26. 10.1016/S0190-7409(01)00141-4

[16] Cooper CE McLanahan SS Meadows SO Brooks-Gunn J. Family structure transitions and maternal parenting stress. J Marriage Fam (2009) 71(3):558–74. 10.1111/j.1741-3737.2009.00619.x 20046951 PMC2743274

[17] Targosz S Bebbington P Lewis G Brugha T Jenkins R Farrell M Lone mothers, social exclusion and depression. Psychol Med (2003) 33:715–22. 10.1017/S0033291703007347 12785473

[18] Achenbach T Manual for the Child Behavior Checklist/4-18 and 1991 Profile. Burlington, VT: Department of Psychiatry, University of Vermont (1991).

[19] Ackermann BP Brown ED D’Eramo KS Izard CE. Maternal relationship instability and the school behavior of children from disadvantaged families. Dev Psychol (2002) 38(5):694–704. 10.1037/0012-1649.38.5.694 12220048

[20] Cooper CE Osborne CA Beck AN McLanahan SS. Partnership instability, school readiness, and gender disparities. Sociol Educ (2011) 84(3):246–59. 10.1177/0038040711402361 21949448 PMC3178046

[21] Fomby P Cherlin AJ. Family instability and child well-being. Am Sociol Rev (2007) 72(2):181–204. 10.1177/000312240707200203 21918579 PMC3171291

[22] Osborne C McLanahan S Partnership instability and child well-being. J Marriage Fam (2007) 69:1065–83. 10.1111/j.1741-3737.2007.00431.x

[23] Hahlweg K Richter D. Prevention of marital instability and distress. Results of an 11-year longitudinal follow-up study. Behav Res Ther (2010) 48:377–83. 10.1016/j.brat.2009.12.010 20053393

[24] Kamp Dush CM Amato PR Consequences of relationship status and quality for subjective well-being. J Soc Pers Relat (2005) 22(5):607–27. 10.1177/0265407505056438

[25] Kamp Dush CM Taylor MG Kroeger RA. Marital happiness and psychological well-being across the life course. Fam Relat (2008) 57(2):211–26. 10.1111/j.1741-3729.2008.00495.x 23667284 PMC3650717

[26] Kim HK McKenry PC The relationship between marriage and psychological well-being: a longitudinal analysis. J Fam Issues (2002) 23(8):885–911. 10.1177/019251302237296

[27] Beach SRH Sandeen EE O’Leary KD Depression in Marriage: A Model for Etiology and Treatment. New York: Guilford Press (1990).

[28] Ablow JC Cowan CP Cowan PA Measelle JR Linking marital conflict and children’s adjustment: the role of young children’s perceptions. J Fam Psychol (2009) 23(4):485–99. 10.1037/a0015894 19685984

[29] Amato PR Afifi TD Feeling caught between parents: adult children’s relations with parents and subjective well-being. J Marriage Fam (2006) 68:222–35. 10.1111/j.1741-3737.2006.00243.x

[30] Cina A Bodenmann G Zusammenhang zwischen Stress der Eltern und kindlichem Problemverhalten. [Relationship between parental stress and child problem behavior]. Kindheit Entwickl (2009) 18:39–48. 10.1026/0942-5403.18.1.39

[31] Cummings EM Davies PT Marital Conflict and Children. An Emotional Security Perspective. New York: Guilford (2010).

[32] Heinrichs N Bertram H Kuschel A Hahlweg K Parent recruitment and retention in a universal prevention program for child behavior and emotional problems: barriers to research and program participation. Prev Sci (2005) 6:275–86. 10.1007/s11121-005-0006-1 16075192

[33] Sanders MR Development, evaluation, and multinational dissemination of the triple-P-positive parenting program. Annu Rev Clin Psychol (2012) 8:345–79. 10.1146/annurev-clinpsy-032511-143104 22149480

[34] Winkler J Die Messung des sozialen Status mit Hilfe eines Index in den Gesundheitssurveys der DHP. [Measuring the social status using the heath survey index of the DHP]. In: Ahrens W Bellach BM Jöckel KH, editors. Messung soziodemographischer Merkmale in der Epidemiologie Schriften des Robert Koch-Institut 1/98. Berlin: Robert Koch-Institut (1998). p. 69–74.

[35] Statistisches Bundesamt. Alleinerziehende in Deutschland – Ergebnisse des Mikrozensus 2009 [Single Parents in Germany – Results of the Microzensus 2009]. Wiesbaden: Statistisches Bundesamt (2010).

[36] Sharpley CF Rogers HJ Preliminary validation of the abbreviated Spanier Dyadic Adjustment Scale: some psychometric data regarding a screening test of marital adjustment. Educ Psychol Meas (1984) 44:1045–9. 10.1177/0013164484444029

[37] Köppe E Glückliche Eltern, liebe Kinder: Auswirkungen von Partnerschaft und psychischer Symptomatik der Eltern auf das Verhalten ihrer Kinder [Dissertation]. Braunschweig, Germany: Technische Universität Braunschweig (2001).

[38] Achenbach TM Rescorla LA Manual for the ASEBA Preschool Forms & Profile. Burlington: Department of Psychiatry, University of Vermont (2000).

[39] Arbeitsgruppe Deutsche Child Behavior Checklist. Elternfragebogen für Klein- und Vorschulkinder (CBCL 1 1/2-5). [German Translation of the Child Behavior Checklist (CBCL/1 1/2-5)]. Köln: Arbeitsgruppe Kinder-, Jugend- und Familiendiagnostik (KJFD) (2000).

[40] Arbeitsgruppe Deutsche Child Behavior Checklist. Elternfragebogen über das Verhalten von Kindern und Jugendlichen; Deutsche Bearbeitung der Child Behavior Checklist (CBCL/4-18). Einführung und Anleitung zur Handauswertung. Köln: Arbeitsgruppe Kinder-, Jugend- und Familiendiagnostik (1998).

[41] Lovibond PF Lovibond SH. The structure of negative emotional states: comparison of the Depression Anxiety Stress Scales (DASS) with the Beck Depression and Anxiety Inventories. Behav Res Ther (1995) 33:335–43. 10.1016/0005-7967(94)00075-U 7726811

[42] Statistisches Bundesamt. Definition Alleinerziehende. [Definition Single Parents]. Wiesbaden: Statistisches Bundesamt (2009).

[43] Carlson MJ Family structure, father involvement, and adolescent behavioral outcomes. J Marriage Fam (2006) 86:137–54. 10.1111/j.1741-3737.2006.00239.x

[44] Markman HJ Floyd FJ Stanley SM Lewis HC Prevention. In: Jacobson NS Gurman AS, editors. Clinical Handbook of Marital Therapy. New York: Guildford Press (1986). p. 173–95.

[45] Hahlweg K Markman HJ Thurmaier F Engl J Eckert V Prevention of marital distress: results of a prospective longitudinal study. J Fam Psychol (1998) 12(4):543–56. 10.1037/0893-3200.12.4.543

[46] Halligan SL Murray L Martins C Cooper PJ. Maternal depression and psychiatric outcomes in adolescent offspring: a 13-year longitudinal study. J Affect Disord (2007) 97:145–54. 10.1016/j.jad.2006.06.010 16863660

